# Supplementary material for: Measles and Rubella Incidence and Molecular Epidemiology in Senegal: Temporal and Regional Trends during Twelve Years of National Surveillance, 2010–2021
Source: Viruses. 2022 Oct 17;14(10):2273. doi: 10.3390/v14102273 (PMC9607477; doi:10.3390/v14102273)
Supplement: Supplementary file 1 [file viruses-14-02273-s001.zip › viruses-1870807-supplementary.pdf]

Supplementary Material

Measles and Rubella Incidence and Molecular Epidemiology in Senegal: Temporal and Regional Trends during Twelve Years of National Surveillance, 2010–2021

Mamadou Malado Jallow [1], Bacary Djilocalisse Sadio [1], Marie Pedapa Mendy [1], Sara Sy [1], Amary Fall [1], Davy Kiori [1], Ndiendé Koba Ndiaye [1], Yague Diaw [1], Déborah Goudiaby [1], Boly Diop [2], Mbayame Ndiaye Niang [1], Elizabeth J. A. Fitchett [3] and Ndongo Dia [1,*]

[1] Département de Virologie, Institut Pasteur de Dakar, Dakar BP 220, Senegal
[2] Division Surveillance Epidémiologique et Riposte Vaccinale du Ministère de la Santé et de L'action Sociale, Dakar BP 220, Senegal
[3] Clinical Research Department, London School of Hygiene and Tropical Medicine, London WC1E 7HT, UK
* Correspondence: ndia@pasteur.sn; Tel.: +221-33-839-92-25

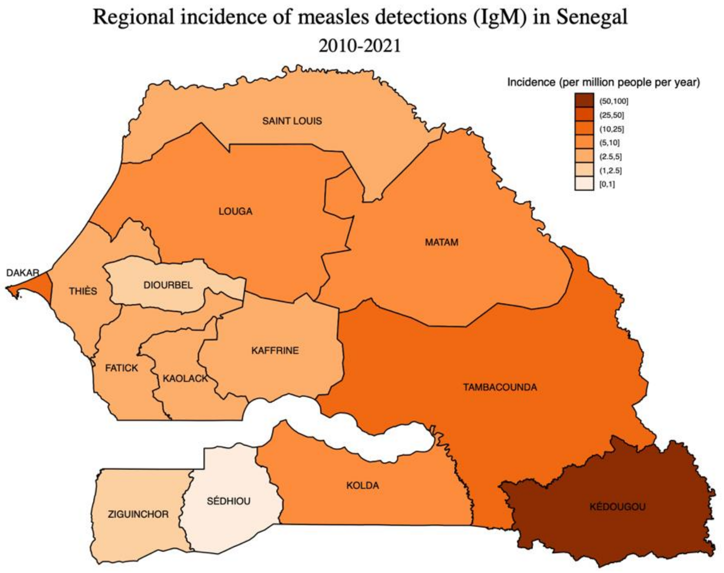

(**A**)

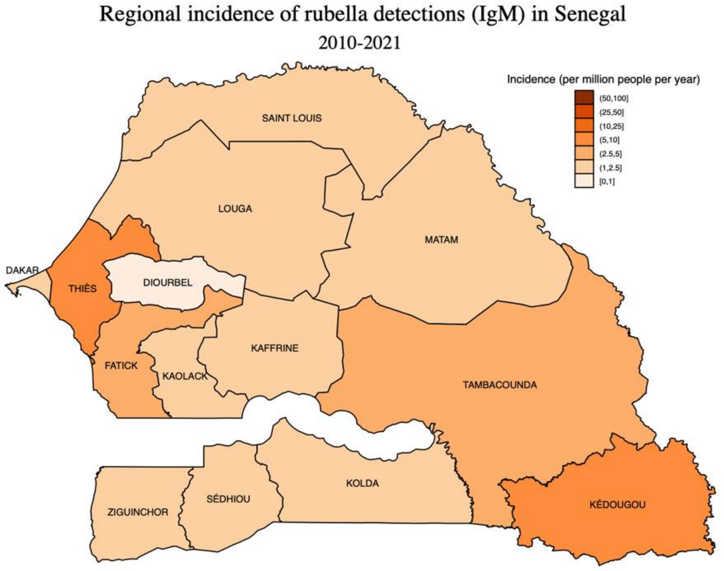

(**B**)

Figure S1. Overall incidence of measles (**A**) and rubella (**B**) IgM detections by region during the whole surveillance period (2010-2021). Incidence estimates were calculated using the median region-specific population denominators between 2010 and 2021. Population data sourced from Agence Nationale de la Statistique et de la Démographie (ANSD): www.ansd.sn.

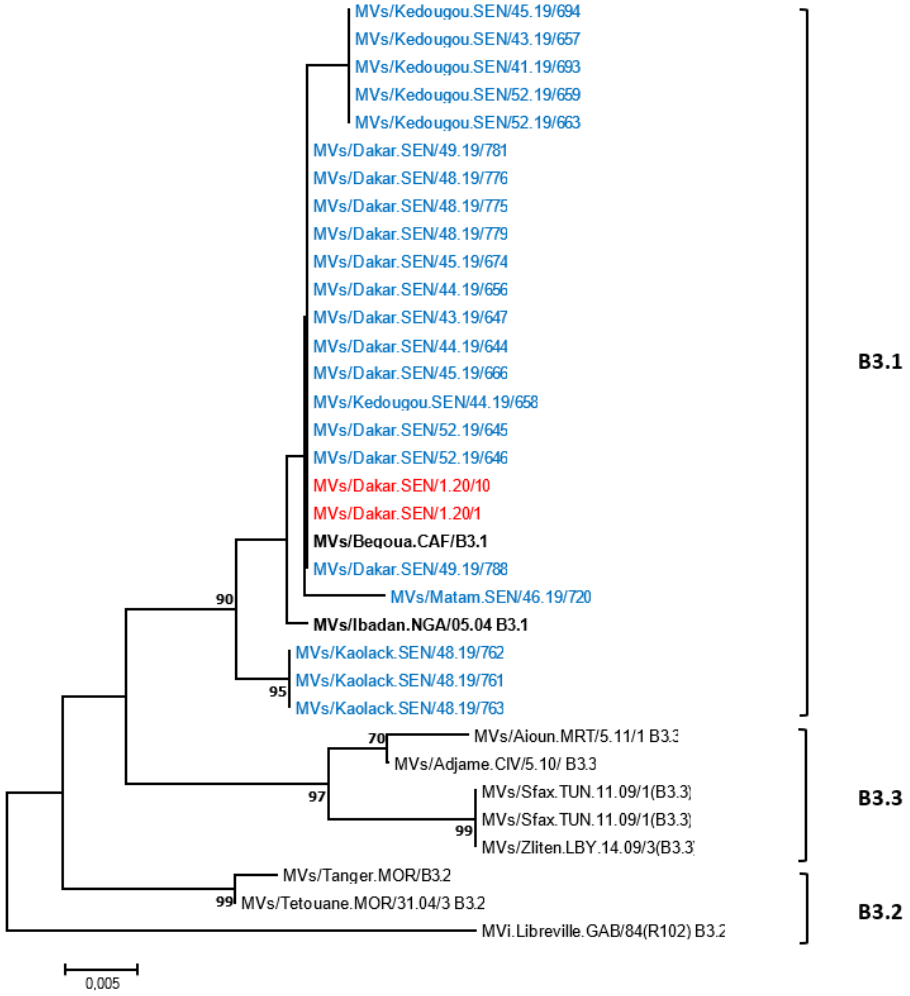

Figure S2. Phylogenetic relationships of Senegalese B3 strains with the sequences of different B3 sub-genotype retrieved from the GenBank database. The tree was constructed based on the 450 nucleotides coding the C-terminal end of the nucleoprotein gene using the neighbor-joining method under the Kimura 2-parameter model, as implemented in MEGA 7 software. The robustness of the nodes was tested with 1000 bootstrap replications and bootstrap support values greater than 70 are shown at the nodes. Senegalese strains are denoted with blue (sequences of 2019) and red (sequences of 2020) colors. Scale bar indicates nuclelotide substitutions per site.
